# Looking Time Predicts Choice but Not Aesthetic Value

**DOI:** 10.1371/journal.pone.0071698

**Published:** 2013-08-16

**Authors:** Eve A. Isham, Joy J. Geng

**Affiliations:** Department of Psychology, Center for Mind and Brain, University of California, Davis, California, United States of America; University of Bath, United Kingdom

## Abstract

Although visual fixations are commonly used to index stimulus-driven or internally-determined preference, recent evidence suggests that visual fixations can also be a source of decisional bias that moves selection toward the fixated object. These contrasting results raise the question of whether visual fixations always index comparative processes during choice-based tasks, or whether they might better reflect internal preferences when the decision does not carry any economic or corporeal consequences. In two experiments, participants chose which of two objects were more aesthetically pleasing (Exp.1) or appeared more organic (Exp.2), and provided independent aesthetic ratings of the stimuli. Our results demonstrated that fixation parameters were a better index of choice in both decisional domains than of aesthetic preference. The data support models in which visual fixations are specifically related to the evolution of decision processes even when the decision has no tangible consequences.

## Introduction

Aesthetic experiences are highly subjective: what one person finds beautiful, another may find dreadful. These idiosyncratic differences occur because aesthetic preferences are shaped not only by visual features such as symmetry [1], but also by our personal knowledge and experiences [2], [3], [4]. Thus, when choosing which of two objects we find more aesthetically pleasing, one might expect that the object with higher personal value would always be selected. However, recent evidence has shown that choices can be biased by information unrelated to how much we value an item. For example, Krajbich et al. [5] found that visual fixations biased snack choice independently of the individual’s original preference: the longer an item was fixated, the more likely it was to be chosen.

The finding that visual fixations played an active role in biasing decisions, potentially in opposition to personal preferences, is particularly interesting within the context of aesthetic choices because looking times have long been used as a measure of preference ( [6], [7]; but see [8] for counter evidence). For example, in the preferential looking paradigm [9], [10], [11] objects that are novel, salient, or otherwise preferred, elicit more fixations and longer looking durations [12]. In these studies, fixations are generally considered to be motor epiphenomena of underlying mental representations. This view of visual fixations as a passive index of personal preference stands in contrast to studies of decision-making in which fixations are not mere reflections of preference, but active biases of the final choice decision (e.g., [5], [13], [14]). One reason that aesthetic choices might differ from that of other decisions is that they do not lead to any clear benefits or consequences, which are a condition of most choice tasks [15].

Aesthetic choices therefore exist as an interesting boundary condition. If visual fixations actively contribute to decision-making, irrespective of the nature of the choice, then we would expect longer fixations on subsequently chosen objects even when it was rated as being less preferred than alternatives. However, if aesthetic decisions are arrived at through processes dominated by preference, then visual fixations should index preference over choice. Our results show that visual fixations reflect comparative processes during choice decisions, irrespective of the nature of the decision and even in opposition to aesthetic preference. The results suggest that the use of visual fixations must consider the context of the paradigm in order to avoid confounding mechanisms of choice with preference.

## Experiment 1

The goal of this experiment was to determine if visual fixations more closely reflected the aesthetic preference of objects viewed in isolation or comparative processes during a two alternative-forced-choice decision.

### Method

#### Participants

Twenty volunteers consented and participated (9 females; 17 right handed, age range 18–24). In this and all subsequent experiments, participants had normal or corrected-to-normal vision.

#### Ethics statement

Written informed consent was obtained, and the study complied with the guidelines and was approved by the Internal Review Board of the University of California, Davis.

#### Materials and procedure

**Figure 1:**
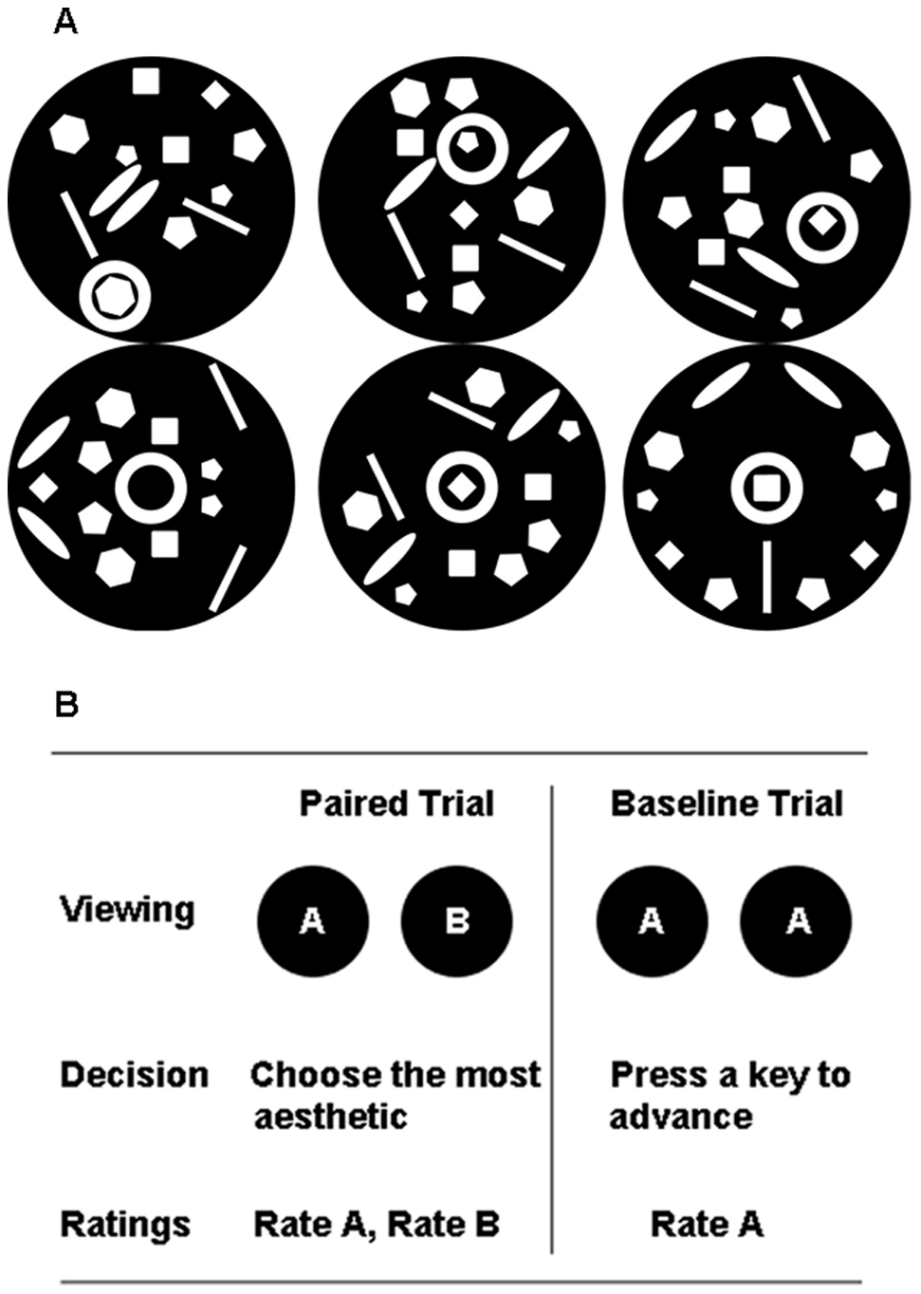
A) Example of a stimulus family. Each object had identical features and overall perceptual luminance as its siblings. Three objects within each family were symmetrical on at least one axis. B) Illustration of the task structure. Two objects were presented during the viewing period. When ready participants first made a choice of which was more aesthetically pleasing (during Paired trials) or pressed a key to advance if the two objects were identical (during baseline trials). Upon keypress, the objects disappeared and subjects were prompted for an aesthetic rating of each object.

Stimuli were novel black-and-white patterns spanning 4.3° in diameter (Figure 1A). To ensure that there was no pre-exposure bias (e.g., famous faces or favorite candy bar), the stimuli were created especially for the experiment. Two types of trials were intermixed and presented in random order (Figure 1B). The 63 “paired” trials consisted of two patterns that had the same number of elements but were differently arranged. Each object appeared in a paired trial only once, resulting in one pair with two symmetrical objects, one with two asymmetrical objects, and one mixed. The center-to-center distance between the two objects were 3°, 6°, or 9° of visual angle. Distance and pair symmetry were used to prevent stereotyped eye-movements and control for stimulus effects on aesthetic judgments, but were unimportant for our primary analyses. The task on paired trials was to choose which of the two images was more aesthetically pleasing by pressing the “z” and “/” buttons for the left and right objects, respectively. The stimuli remained visible until a manual response, after which the two images disappeared and participants were asked to rate the aesthetic value of the two items on a Likert scale of 1 to 9, beginning with the left item first.

During “single-item” trials, only one pattern appeared (126 trials total), but in duplicate bilaterally to be visually identical to paired trials [16]. Participants pressed one of the two buttons to advance the trial and assign a single aesthetic value to the stimulus. The critical difference between paired and single-item trials was that paired trials required an evaluative comparison of two stimuli whereas decisions in the single-item trials were isolated from any comparative processes. The aesthetic rating on single-item trials therefore reflected the *unbiased* preference for a stimulus and we refer to these the “baseline” ratings [17], [18], [19]. Eye-data were acquired using the Eyelink II (SR Research) from the left eye at 500 Hz.

### Results

#### The relationship between value and choice

In order to test whether fixations were indicative of aesthetic choice or preference, it was first necessary to demonstrate that the two measures were imperfectly correlated. In the same fashion as the analysis by Krajbich et al. (2010), we calculated the probability of the left item being chosen as a function of the difference in aesthetic ratings between the left and the right items shown during the paired trials. The difference scores were calculated by subtracting the rating for the right-sided stimulus from the left-sided stimulus, and binning values into five categories: −2 and less, −1, 0, 1, 2 and greater. Difference scores were calculated for both the ratings from the baseline and paired trials. Note that in paired trials, the ratings followed the explicit choice whereas in baseline trials, there were two identical objects and no explicit choice; the baseline ratings could not have been influenced by any immediately preceding choice behaviors.

**Figure 2:**
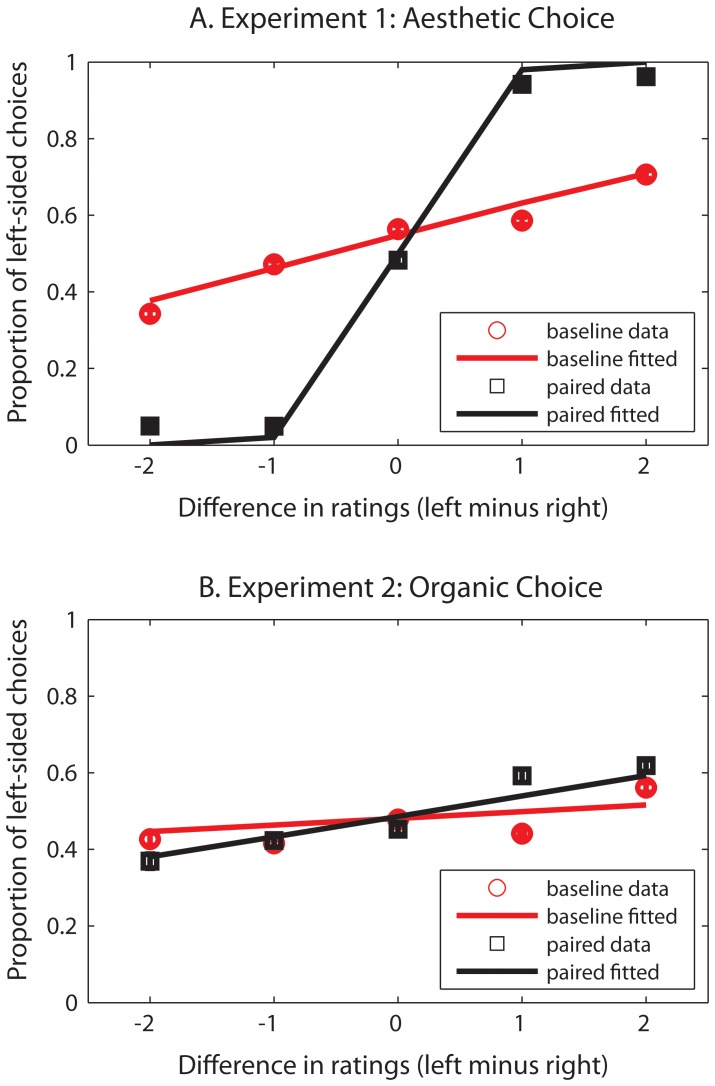
Proportion of trials when the left item was chosen as a function of its difference in rating from the right-sided item. Ratings were taken from baseline trials (red circles), which were temporally independent of the choice decision and from the paired trials (black squares), which immediately followed the choice decision. Error bars are standard error of the mean. Lines are average model fits. A) Experiment 1. B) Experiment 2.

To quantify the relationship between aesthetic choices and aesthetic ratings, the proportion of left choices (bounded by 0 and 1) were modeled using a logistic function given by, y = exp(A*(x−B))/(1+exp(A*(x−B))), where A is the slope and B is the intercept of the function (Figure 2). We fit the model to data from each subject, separately for difference scores calculated from baseline trials and from the paired trial. The A parameter value from one subject in the baseline data was a clear outlier, being more than an order of magnitude greater than any other subject’s value (i.e., a value of 10.1 where all other values were less than 1.2) and was therefore removed in calculation of group parameters.

In the baseline data there was a monotonic increase in the likelihood of choosing the higher rated item as the relative rating increased (mean A = .38; average model fit parameters: rmse = .13, R^2^ = .50). However, on paired trials, the data were well fit by a logistic function with a large slope (mean A = 6.71; rmse = .05, R^2^ = .99), which reflected categorically higher ratings for the chosen item. The A parameters were significantly different from each other using a paired t-test, t(18) = 5.7, p<.0001. The results indicated that paired ratings were highly influenced by the preceding choice, which occurred on the same trial (i.e., the “choice-supportive bias” in which the chosen object is rated more highly to avoid cognitive dissonance; [20], [21], [22]). This confirmed that the baseline ratings were the more unbiased measure of aesthetic preference. We therefore use this baseline rating in subsequent analyses of item preference because it was more independent of choice related cognitive processes engaged on paired trial.

#### The relationship between visual fixations, choice, and value

We next entered the proportion of time spent looking at each object in a paired trial into a 2 *choice* (chosen, unchosen)×3 *relative baseline rating* (higher, lower, same) repeated measures ANOVA. An object’s *relative baseline rating* was classified as higher, lower or the same if the object’s baseline rating was greater than, less than, or equal to its pair counterpart, respectively.

**Figure 3:**
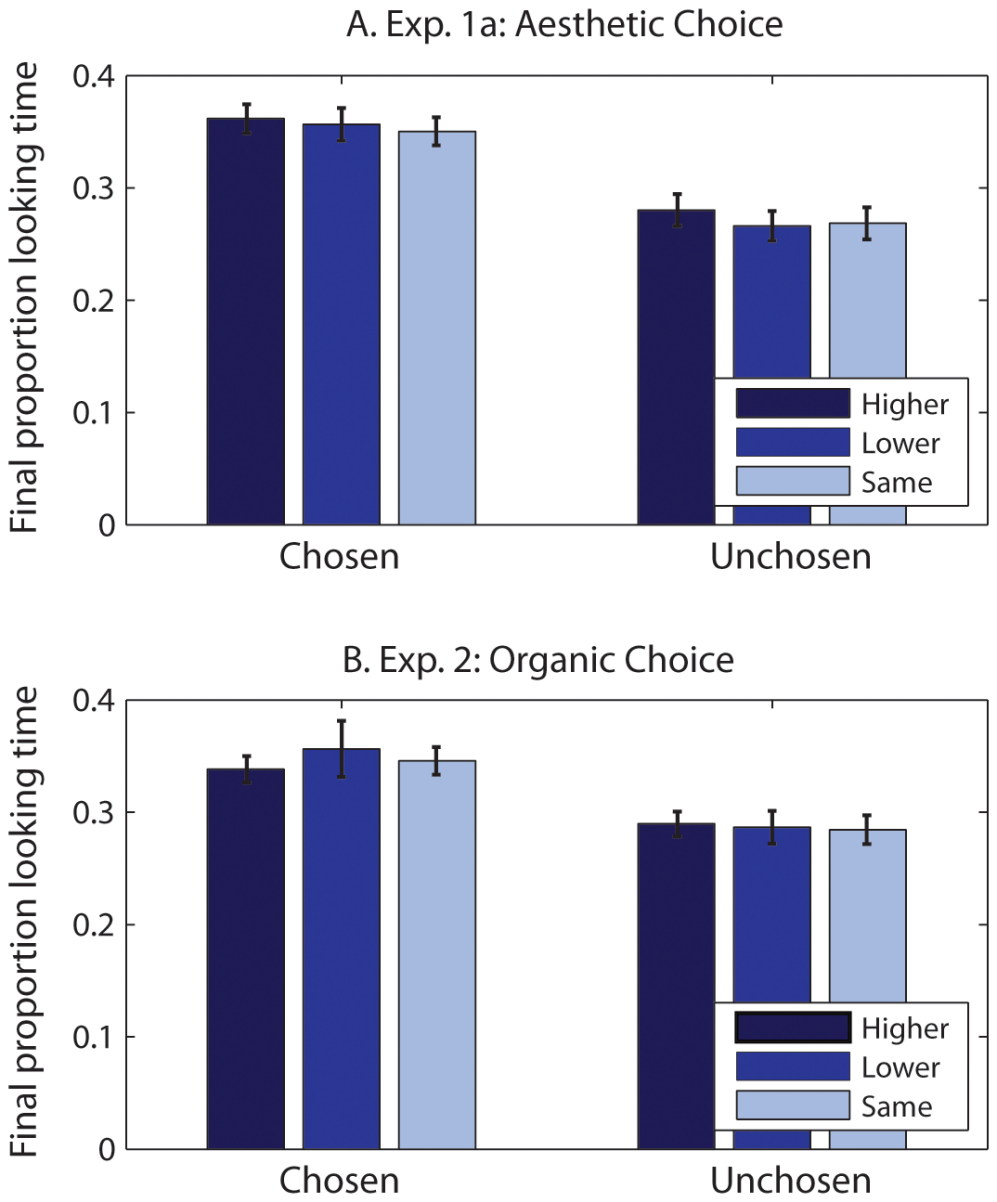
Final proportional looking times for chosen and unchosen items for trials in which the chosen item had a higher baseline rating (dark blue), the chosen item had a lower baseline rating (medium blue), and the two items had the same baseline rating (light blue). A greater proportion of the total fixation duration was on the chosen item across all differences in baseline ratings.

There was a significant main effect of choice such that the chosen object (M = .36, SE = .01) was fixated for longer compared to the unchosen object (M = .27, SE = .01), F(1,19) = 128.75, p<.001, η^ 2^ = .87). There was no main effect of the relative baseline rating, F(2,38) = 2.69, p = .08, η^2^ = .12, nor an interaction, F(2,38) = .59, p = .56, η^ 2^ = .03. Thus, proportional fixation durations were clearly longer for the subsequently chosen object (Figure 3). Fixations were not significantly affected by the relative baseline ratings, but note that the statistic shows marginal significance; this suggests that the relative baseline ratings may have had a smaller effect on performance.

**Figure 4:**
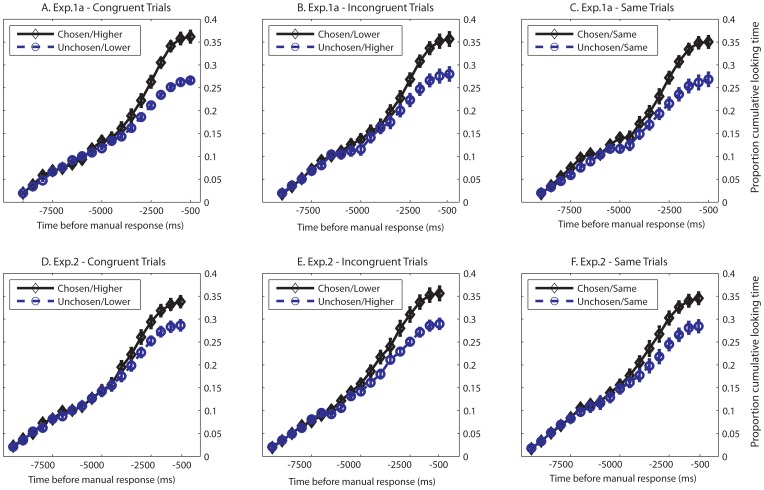
Proportional cumulative fixation durations time-locked to the manual response indicating choice decision. Trials were analyzed separately based on the congruency between choice and baseline aesthetic ratings. Figures 4A and 4D contain congruent trials (chosen item also had higher baseline rating), for Experiments 1a and 2, respectively. Figures 4B and 4E contain incongruent trials (chosen item had lower baseline rating). Figures 4C and 4F contain trials with objects given the same baseline rating. Looking times diverged statistically substantially earlier than the explicit manual response (3500ms before the aesthetic decision, and 2500ms before the organic decision).

To identify the point at which fixation durations became significantly longer for the subsequently chosen object, we calculated the cumulative proportional fixation duration over 500 ms bins, time-locked to the choice response (Figure 4). We did this separately for trials in which the choice was congruent with the rating (i.e., the chosen item was also had a higher aesthetic rating), incongruent with the rating (i.e., the item with the lower baseline rating was chosen), and for trials in which the ratings were the same. On average, there were 32.2 (SD = 7.81) trials in the congruent condition, 16.85 (SD = 5.89) in the incongruent condition, and 13.9 (SD = 5.48) in the same condition. In this way, we were able to determine whether the pattern of cumulative looking times were similar for the chosen vs. unchosen objects across rating congruency.

In order to test for significant differences between the cumulative fixation durations on the chosen vs. unchosen object, we conducted sequential t-tests, beginning from the data bin nearest to response (i.e., 500 to 0 ms *before* manual response). If the difference at a time bin was significant, a t-test of the next time bin (e.g., 1000 to 500 ms before manual response) was conducted. This analysis demonstrated that the proportional looking duration of the chosen item was significantly longer than the unchosen item beginning at 3500 ms prior to the actual manual response when two measures were congruent, t(19) >3.99, p<.001 with Bonferroni correction for seven comparisons, at which point the difference was no longer statistically significant. On incongruent trials where the chosen object was given a lower baseline rating (M = 16.85, SE = 5.89 trials per participant) the cumulative fixation duration on the chosen object was significantly longer than the unchosen object beginning from 3000 ms before the manual response, t(19) = 2.99, p<.05, with Bonferroni correction for six comparisons. On same-rating trials, the difference in the cumulative looking time also began as early as 3000 ms before the response, t(19) = 3.58, p<.05, with Bonferroni correction for six comparisons.

In addition to total fixation durations, we also examined whether the last fixation was a better indicator of the subsequently chosen item, or the more preferred item. This was motivated by literature showing that final fixations vary with choice and aesthetic preference [5], [19]. Overall there was a greater proportion of trials in which the final fixation was on the chosen (M = .70, SE = .02) than the unchosen object (M = .30, SE = .02), F(1,19) = 76.69, p<.001, η^2^ = .80. However, this effect was mediated by aesthetic ratings: the conditional probability of the last fixation being on the chosen item as a function of its relative baseline rating (i.e., rated higher, lower, or the same as the unchosen object) was significant, F(2,38) = 14.83, p<.001, η^2^ = .44 (M-higher = .80, SE = .03, M-lower = .56, SE = .04, and M-same = .74, SE = .04). Post-hoc paired t-tests revealed that amongst the chosen objects, those with lower ratings were less likely to be looked at last compared to those with higher or same ratings, both t(19) = 3.78, p<.001, d = 1.01 There was no difference between those rated higher and the same, t(19) = 1.51, p>.14.

### Discussion

The goal of Experiment 1a was to examine if eye movement parameters (i.e., fixation duration and last fixation) would serve as indices of the choice process and/or aesthetic preference. We observed that fixation durations and final fixation corresponded to the subsequent explicit choice: when choice and aesthetic ratings differed, these parameters predicted choice and not ratings. Moreover, we observed that the cumulative fixation time on the subsequently chosen object was reliably longer on the chosen object approximately 3000 ms before the manual button press that indicated that choice. These results suggested that aesthetic choices engage similar mechanisms as decisions in other cognitive or corporeal domains [5]. However, it may be that the choice decision masked the effect of aesthetic ratings because they were based on the same dimension. We therefore further tested the relationship between eye movement parameters and preference (i.e., indicated by relative baseline ratings) in tasks that did not involve simultaneous rating and choice aesthetic judgments. In Experiment 1b and Experiment 2, the choice dimension was dissociated from the judgment of aesthetic preference.

## Experiment 1b

In Experiment 1a, ratings and choice were based on an aesthetic judgment. It was possible that effects of choice simply masked those of ratings because they were in the same dimension. To address this question, we conducted an identical experiment, but now removed the choice component. If the influence of preference on looking durations were masked in Experiment 1a by the choice decision, we should see a difference in the cumulative looking times as a function of aesthetic ratings in this experiment when no choice was required.

### Methods

#### Participants

Twenty volunteers participated (11 females; 17 right handed, age range 18–24).

#### Materials and procedure

The materials and procedure were identical to Experiment 1a except that the explicit choice portion of each trial was removed. On each trial, the stimuli were terminated when participants pressed a button to indicate their readiness to provide paired aesthetic ratings.

### Results

Similar to the Experiment 1a, we first compared the total proportion of looking time on each of the paired objects as a function of their baseline rating. Because the decision component was removed, the looking time was analyzed as a function of the relative baseline ratings. The cumulative looking times for the higher-rated item (M = .51, SE = .01) was not statistically different from the lower-rated item (M = .49, SE = .01), t(19) = 1.0, p>.05.

Given that the baseline and paired ratings were imperfectly correlated (average R = .30, SD = .21), a second analysis was conducted using the aesthetic ratings from the paired trials. Note that this was not possible in Experiment 1a because the paired ratings were contaminated by the choice (i.e., the “confirmation bias”; see above). Still, the total proportional looking durations were not significantly different for the higher- and lower-rated items, t(19) = .24, p>.5 (object rating higher : M = .50, SE = .01; lower: M = .50, SE = .01). Looking times did not reflect aesthetic ratings when these ratings were made in the absence of choice.

These results suggest that longer fixation observed in Experiment 1a was driven more by the decision process rather than by aesthetic preference. Furthermore, the mean RT per trial was not statistically different from Experiment 1a, t(38) = .68, p>.4; M = 3553.35 ms, SE = 360.77, suggesting that the two tasks required comparable processing times.

### Discussion

The decision and rating processes in Experiment 1a were both based on aesthetic judgments. Due to this overlap, it was difficult to rule out contributions from aesthetic ratings on eye movement parameters. Thus, in Experiment 1b we dissociated the choice and rating processes by asking participants to only rate items based on aesthetic value. Critically, the choice component was removed. Nevertheless, the eye movement parameters still did not correlate with measurements of preference rating. This provides further evidence that the eye parameters did not reflect preference.

## Experiment 2

To further test the relationship between fixations, choice and aesthetic preference, we next dissociated the dimensions of the choice decision and preference by asking subjects to choose which of the two objects was more “organic” looking, but to rate each based on aesthetic preference. To confirm the results from Experiment 1a which showed stronger correlations between the choice process and eye parameters and not between aesthetic preference and eye parameters, it would be necessary to show in Experiment 2 that the correlation holds even for non-aesthetic based choice. Thus, in this experiment, the participants were asked to choose on the ‘organic’ dimension. We predicted that looking time would be longer for the chosen item, and that the chosen item would be fixated last. We did not expect parameters to vary with aesthetic ratings.

### Methods

#### Participants

Twenty new volunteers consented and participated (4 females; 13 right handed, age range 18–24) in the study.

#### Materials and procedure

The materials and procedure were identical to Experiment 1 with the exception that participants were now asked to choose which of the two objects looked more “organic.” Procedures for acquiring baseline and paired aesthetic ratings were identical to Experiment 1a.

### Results

#### The relationship between value and choice

As in Experiment 1a, we first modeled the proportion of left choices as a function of baseline and paired trial ratings using a two parameter logistic function (Figure 2b). The slope parameters from both models were relatively shallow (baseline: mean A = .15; rmse = .16, R^2^ = .4; paired: mean A = 1.88; rmse = .17, R^2^ = .56), suggesting a weak relationship between the item chosen as being more organic and their respective aesthetic ratings. The difference in A parameters was marginally significant, t(19) = 1.77, p = .092). Interestingly in contrast to Experiment 1, the paired ratings were less influenced by the same-trial choice now that the choice was made on a different stimulus dimension (organic quality vs. aesthetic preference).

#### Visual fixations, choice, and value

The total proportion of looking time on paired trials was entered into a 2 choice (chosen, unchosen)×3 relative baseline rating (higher, lower, same) repeated-measures ANOVA (Figure 3). There was a main effect of choice such that the chosen object (M = .35, SE = .01) was fixated longer than the unchosen object (M = .29, SE = .01), F(1,19) = 26.06, p<.001, η^2^ = .58. There was no significant effect of rating, F(2.26) = 1.12, p = .34, η^2^ = .06, nor an interaction between the two, F(2,38) = 1.16, p = .32, η^2^ = .06. This result was consistent with Experiment 1a and suggested that looking durations reflected comparative processes involved in choice decisions and not aesthetic preference.

Furthermore, as in Experiment 1a, we analyzed the time series analysis data separately for trials in which the chosen item was congruent, incongruent, or the same as the one with the higher baseline aesthetic rating. Note that in this experiment, congruency between the organic choice and ratings were on different dimensions, but still referred to the convergence (or divergence) of choice with the baseline aesthetic rating. For example, the two dimensions would be congruent if the item chosen as being more organic was also given a higher baseline aesthetic rating.

The data were analyzed separately for trials in which the choice and ratings were congruent (M = 26.10 trials, SD = 7.13), incongruent (M = 22.45 trials, SD = 6.57), or the same (M = 14.30 trials, SD = 4.45). The difference in proportional looking time for both the congruent and incongruent trials was statistically significant beginning at 2500 ms prior to the response, both t(19) >3.28, p<.05, with Bonferoni correction for five comparisons. The difference in proportional looking time for trials with the same baseline ratings was statistically significant starting at 3000 prior to response, t(19) = 3.31, p<.05, with Bonferoni correction for six comparisons. As with Experiment 1, these data suggest that the cumulative looking times reflected decision processes and not aesthetic ratings. The overall RT data from Experiment 1a (M = 3770.32 ms, SE = 440.62) was not statistically different from Experiment 2 (M = 5211.86, SE = 828.46), t(38) = 1.53, p>.1.

The last fixation data were also similar to Experiment 1a such that the proportion of final fixations on the chosen object (M = .60, SE = .03) was greater than those on the unchosen object (M = .40, SE = .03), F(1,19) = 12.89, p<.005, η^2^ = .40. However, unlike Experiment 1a, this effect was not modulated by the aesthetic ratings of the chosen item, F(2,38) = 1.15, p>.30, η^2^ = .06, (proportion of final fixations on the chosen object as a function of its rating: higher: M = .64, SE = .04; lower: M = .56, SE = .04; same: M = .61, SE = .04).

### Discussion

Overall, the results are consistent with those of Experiment 1a. These findings provide further evidence that visual fixations were reliably related to arbitrary subjective decisional processes and not to the aesthetic preferences.

## General Discussion

Visual fixations have a long history of being used to assess internal mental representations, and among these are studies that have used looking times as proxy for preference [6], [20]. However, work in the decision-making literature has recently suggested that visual fixations are not simply a consequence of internal representations, but may also reflect processes involved in comparing two or more items during decision making. In the current study, we sought to understand the metric of information carried by visual fixations during decision tasks that do not result in any tangible consequences (cf. buying a car, choosing a snack). These decisions are particularly interesting because they serve as boundary conditions between choices (which normally carry consequences) and personal preferences (which may be unaffected by decision processes). The purpose was to determine whether measurements of visual fixations should be understood in terms of reflecting preferences or cognitive processes related to the comparison of objects that occur during choice decisions.

In Experiment 1, we found that the object chosen as more aesthetically pleasing was not always given a higher aesthetic rating when viewed alone. This result was similar to previous findings in which choice decisions were influenced by factors beyond the personal value of objects [5]. However, given that aesthetic choices differ from other decision-making situations in that no consequences or tangible benefits occur [15], one might have expected that only preferences should drive visual fixations. Instead we found that the chosen object was looked at longer than the unchosen item both when the ratings were congruent with the choice as well as when they were incongruent; moreover reliable differences began about 3000–3500 ms prior to the actual manual response, suggesting that the final choice outcome could be predicted before the manual button press was made to indicate choice. These early differences in looking time prior to the explicit decisional response are intriguing as they might serve as a time-sensitive pre-decisional indicator of choice better than verbal responses [21], [22]. Overall, the general results corroborate that of Krajbich et al. [5] illustrating that fixation durations serve as an ongoing indicator of the comparison processes and not just the instantaneous value of the compared items. Thus aesthetic decisions appear to rely on mechanisms similar to other types of value-based [5] and perceptual decision-making [23], [24], [25].

To further test the relationship between fixation parameters and choice processes, dimensions of choice and preference were dissociated in Experiment 2: participants chose which of the two objects looked more “organic.” Organic choices are completely unrelated to aesthetic preferences, but similar to an aesthetic choice; an “organic” decision is arbitrary and devoid of any direct consequences (e.g., corporeal or financial). We found that, consistent with Experiment 1, participants spent more time fixating the object chosen as being more “organic.” There were no reliable effects of aesthetic ratings on fixation durations.

Overall, the current data suggest that preferences play little role in controlling visual fixations when a choice decision is being made. However, it is possible that this may have been partly due to the fact that our simple geometric stimuli lacked emotional content [13]. Further work is necessary to test whether more complex and emotional stimuli would produce a stronger effect of aesthetic preference on patterns of fixation.

In conclusion, our data demonstrated that cumulative fixation durations indexed the evolution of comparative processes that preceded a choice decision, even when the choice was entirely subjective. The results enhance understanding of how distinct parameters of visual fixations are related to preferences and choice processes [5], [12]. These findings are a novel extension to the decision-making literature in showing that even aesthetic and “organic” judgments for which there are no clear benefits or consequences involve similar comparative processes that precede other types of decisions [5], [15]. Moreover, our findings suggest that visual fixations should be interpreted with caution when used as a metric of preference in that patterns may depend upon whether the alternatives are being compared.
